# Impact of WHO multimodal strategy on hand hygiene in a Tunisian hospital: quasi-experimental study

**DOI:** 10.3389/fpubh.2025.1724856

**Published:** 2026-01-15

**Authors:** Hajer Hannachi, Mohamed Mouadh Ben Fradj, Tasnim Meddeb, Donia Ben Hassine, Sana Rouis, Raja Ghzal, Elyes Turki, Latifa Merzougui, Dhekra Chebil

**Affiliations:** 1 Faculty of Medicine of Sousse, University of Sousse, Sousse, Tunisia; 2 Preventive and Community Medicine Department, University Hospital Ibn Eljazzar, Kairouan, Tunisia; 3 Infectious Disease Department, University Hospital Ibn Eljazzar, Kairouan, Tunisia; 4 Emergency Medicine Department, University Hospital Ibn Eljazzar, Kairouan, Tunisia; 5 Forensic Medicine Department, University Hospital Ibn Eljazzar, Kairouan, Tunisia

**Keywords:** compliance, hand hygiene [MeSH], intervention, knowledge, multimodal strategy

## Abstract

**Introduction:**

The World Health Organization (WHO) multimodal strategy is a globally recognized framework designed to improve hand hygiene (HH) knowledge and compliance among healthcare workers (HCWs). Aim: Assess efficacy of WHO multimodal strategy on healthcare workers’ knowledge and compliance on hand hygiene in a teaching hospital in Tunisia.

**Methods:**

A quasi-experimental study with a pre- and post-intervention design was conducted. Baseline data were collected over 4 weeks, followed by a 3-month intervention period and a 4-week post-intervention assessment. To assess knowledge, a Validated WHO questionnaire was used on pre- and post-intervention (scores: 0–100%). To assess HH compliance, the WHO “Five Moments” framework observation was used. Chi-square tests evaluated categorical variables. A *p*-value <0.05 was considered statistically significant.

**Results:**

Knowledge of major causes responsible for healthcare associated infections passed from 34 to 40.3% (*p*= 0.19). Awareness of recommended duration of Alcohol-based hand rub or soap and water increased globally (41.8% vs. 49.3%, *p*=0.29). Concerning HH compliance, global rate significantly increased from 19.9 to 23.6% (*p* < 10–3). Whereas, the HH compliance rate with optimal respect of the prerequisites was marked by a slight drop overall (from 37.2 to 35.5%, *p*=0.12), and this drop was particularly noticeable among physicians (from 42.2 to 39.4%).

**Conclusion:**

This study did not conclude on the efficacy of WHO HH strategy on HCWs knowledge. Whereas, a significantly increased HH compliance rate was globally observed.

## 1 Introduction

The primary objective of hand hygiene is to reduce the transmission of healthcare-associated infections (HAIs), a major public health issue that affects up to 15% of hospitalized patients in low-middle income countries (1). Although its effectiveness has been well established since the observations of Semmelweis in 1847, compliance with hand hygiene remains insufficient, with average rates reported at 40% in developed countries and as low as 20% in developing countries (2). This persistent gap, despite numerous efforts, has prompted the World Health Organization (WHO) to propose a multimodal improvement strategy based on five key components: system change, training and education, evaluation and feedback, workplace reminders, and fostering a safety culture within healthcare institutions (3).

In Tunisia, the burden of HAIs remains significant. The NOSO-TUN survey conducted in 2012 reported a prevalence of 7.7% (4), while other studies revealed rates of 8.4% in Sfax (5), 5.4% across nine hospitals in the center-east region (6), and 45% of serious adverse events associated with HAIs in Sousse (7). At Ibn El Jazzar Hospital, a 2010 survey documented a prevalence of 17%, with subsequent annual surveys ranging from 4 to 6.7%. In response to these concerning figures, the Tunisian national hand hygiene promotion program—launched in 2002—was aligned with the WHO multimodal strategy and implemented in several hospitals, notably at Sahloul University Hospital in Sousse since 2015 (8, 9). Within the same framework, the Ibn El Jazzar University Hospital of Kairouan undertook a quasi-experimental study to evaluate the impact of the multimodal hand hygiene strategy on healthcare workers’ knowledge and compliance.

## 2 Method and material

### 2.1 Study design and participants

#### 2.1.1 Design

This was a quasi-experimental, interventional study (before-and-after), designed to evaluate the effectiveness of the WHO multimodal hand hygiene strategy in improving hand hygiene knowledge and practices among healthcare professionals in Ibn El Jazzar Hospital, which is a university hospital composed of four units (surgical: 196 beds; medical and obstetrics-gynecology: 381 beds; psychiatry: 40 beds; diabetology: outpatient consultations), 33 departments, including 20 hospital departments, with a total capacity of 599 beds. The staff consisted of 1,577 employees: 260 medical staff (16.5%), 1,005 paramedical staff (64%), and 169 workers (10.7%).

The primary outcome measure was hand hygiene compliance before and after the intervention. This strategy is based on a multimodal plan consisting of five elements to be implemented simultaneously (3). The secondary outcome was the enhancement of workers’ knowledge about hand hygiene.

#### 2.1.2 Time

Two surveys were conducted at the same time:

- An assessment of healthcare staff knowledge about hand hygiene in pre-intervention (Baseline assessment: March, April, and May 2024) and post-intervention (October and November 2024).
- An assessment of hand hygiene practices through direct observation, both pre-intervention (Baseline assessment: March, April, and May 2024) and post-intervention (October and November 2024).

A resource audit-type study was conducted before beginning the practice audit.

The observation period was communicated in advance to the relevant care units.

#### 2.1.3 Sampling

The study included physicians, nurses, and technicians in direct contact with patients or their environment. Their hand hygiene knowledge and practices were assessed randomly at various times of the day. According to WHO recommendations, sample size estimates indicate that 200 opportunities per observation period and per unit are necessary for a reliable comparison of results (10). In this study, we define the professional category as a unit of reference and at least 200 observations per professional category. We therefore define three categories: Medical (Physicians, Interns/Residents), Paramedical (nurses/technicians, internship students), and Manual Workers.

### 2.2 Data collection instrument

#### 2.2.1 Assessment of healthcare workers’ knowledge

A questionnaire based on the WHO Multimodal Hand Hygiene Promotion Strategy (3) and the WHO Hand Hygiene Manual (10) was used to assess knowledge.

#### 2.2.2 HH compliance measure’s instrument

Data was collected using an audit form adapted from the WHO Hand Hygiene Promotion Project (3). It consisted of two parts: the first grouped general information (department, professional category, date, time), and the second consisted of a grid of nine tables to record, for each observed professional, indications and actions related to hand hygiene opportunities.

### 2.3 Procedures

#### 2.3.1 Assessment of healthcare workers’ knowledge

The investigators, staff from the hospital’s Infection Prevention and Control department, distributed an anonymous questionnaire based on the WHO Multimodal Hand Hygiene Promotion Strategy (3) and the WHO Hand Hygiene Manual (10). The completed questionnaires were then collected by the same investigators from the ward supervisors.

#### 2.3.2 Assessment of compliance to hand hygiene

The assessment of compliance with hand hygiene practices was carried out in all services, excluding operating rooms and administrative departments.

##### 2.3.2.1 Definition of variables

- Observation: It measured how often and when healthcare workers practiced actions of hand hygiene (11).
- Indication for hand hygiene: Are specific situations that require healthcare professionals to comply with hand hygiene practices to prevent the transmission of infections. And defined as below (10).
o Before touching a patient.
o Before an aseptic procedure.
o After a risk of exposure to a biological fluid.
o After touching a patient.
o After touching a patient’s environment.
- Hand hygiene opportunity: A hand hygiene opportunity occurs the moment when healthcare professionals need to perform hand hygiene in response to a specific indication (11).

##### 2.3.2.2 Hand hygiene action as seen by the observer

Hand hygiene observation is based on the systematic association of each action with a defined opportunity. An opportunity is recorded when an indication for hand hygiene is identified, and the corresponding action is noted as positive (performed) or negative (not performed) (10). When documenting hand hygiene compliance data on the observation form, the observer should keep the following in mind:

a) At least one hand hygiene indication must be observed to count it as an opportunity.
b) Each opportunity must correspond to a hand hygiene action.
c) An action can apply to one or more indications.
d) A documented action can be positive (performed) or negative (not performed), provided it corresponds to an opportunity.
e) The observation of a positive action does not necessarily imply the existence of an opportunity.

#### 2.3.3 The intervention

The intervention was based on the WHO’s multimodal strategy (3).

1. Training and education sessions of all hospital healthcare professionals on the prerequisites, techniques, and indications for hand hygiene (HH): specific morning and afternoon sessions were organized per department in a pace of two training sessions in each department, and an overall total of 40 sessions throughout the program. These training courses were provided by two trainers: a preventive and community medicine assistant (AHU) and a family medicine resident, well-trained in the topic.
2. Reminders at the workplace were distributed especially at handwashing stations.
3. Consumables such as hydroalcoholic solution, liquid soap, and hand towels were distributed to departments where a need was identified.
4. Instilling hand hygiene safety culture by raising awareness among healthcare workers on the benefits of HH.
5. feedback on the audit results prior to the intervention.

#### 2.3.4 Indicators’ definition

Compliance measures the percentage of hand hygiene actions performed relative to the total number of hand hygiene opportunities. The formula is as follows:


$$\begin{array}{l} \text{Compliance}\;(\%)=\\ \frac{\text{Number of hand hygiene actions performed}}{\text{Total number of hand hygiene opportunities}}\;x\;100 \end{array}$$

Indication-specific hand hygiene compliance measures the percentage of hand hygiene actions performed based on specific hand hygiene indications. Note that when a single action covers two successive indications observed during the same opportunity, this action is taken into account for each of the indications concerned.

The formula is as follows:


$$\begin{array}{l} \text{Specific compliance}\;(\%)=\\ \frac{\begin{array}{l} \text{Number of hand hygiene actions}\;\\ \text{performed for an indication}\; \end{array}}{\begin{array}{l} \text{Total number of hand hygiene opportunities}\;\\ \text{where this indication occurred} \end{array}}\;x\;100 \end{array}$$

Compliance with prerequisites measures the percentage of hand hygiene opportunities that meet the prerequisites. The assessed criteria evaluated were short nail, jewelry and clothing hygiene.

The formula is as follows:


$$\begin{array}{l} \text{Compliance with prerequisites}\;(\%)=\\ \frac{\text{Number of opportunities with prerequisites}\;\mathrm{met}\;\;}{\text{Total number of hand hygiene opportunities}}\;x\;100 \end{array}$$

The quality performance rate measures the percentage of hand hygiene procedures performed correctly compared to the total number of hand hygiene procedures performed. The quality assessment parameters are: adherence to the recommended duration and to the correct steps The formula is as follows:


$$\begin{array}{l} \text{percentage of good}-\text{quality actions}(\%)=\\ \frac{\begin{array}{l} \text{Number of hand hygiene}\;\\ \text{actions performed correctly} \end{array}}{\text{Total number of hand hygiene actions}}\;x\;100 \end{array}$$

### 2.4 Data analysis

The study data were entered and analyzed using SPSS software, version 20.0.

Qualitative variables were presented using frequencies and percentages. Quantitative data were presented as mean and standard deviation (if normally distributed) or as median and interquartile range (if not normally distributed). Normality was assessed using the Kolmogorov–Smirnov test. Comparisons between qualitative variables were performed using the chi-square test or Fisher’s exact test. *p*-values less than 0.05 were considered statistically significant.

### 2.5 Ethical considerations

This study adhered to strict ethical standards. Formal approval was obtained from the hospital’s local ethics committee (The Ethics Committee of Ibn al Jazzar University Hospital of Kairouan, Tunisia) and the heads of all participating departments. Healthcare personnel were informed of the study’s aim prior to the anonymous observation of hand hygiene actions. Participation in the knowledge questionnaire was voluntary, and all data were collected and analyzed anonymously to protect participant’s confidentiality. These measures ensured the research was conducted with respect for all participants and institutional guidelines.

## 3 Results

### 3.1 Participation rate

Before the intervention, the overall response rate was 41% (300/730), with a higher participation rate among medical staff (57.1%) compared to non-medical staff (34.6%). After the intervention, the response rate increased to 47.2% (345/730), with stable participation among medical staff (57.6%) and a significant improvement among non-medical staff (43%).

### 3.2 Characteristics of participants

Before the intervention, the average age of participants was 31.9 ± 8.3 years (range: 20–59 years). The sample consisted of 300 professionals, with a majority being female (74%), resulting in a sex ratio of 2.8. Less than half of the participants (44%) had received hand hygiene training in the previous three years, while 80.7% reported using alcohol-based hand rubs (ABHR).

After the intervention, the average age of respondents was 28.9 years (range: 20–59 years). Of the 345 participants, 69.3% were female, with a sex ratio of 2.25. The proportion of participants who had received hand hygiene training increased significantly reaching 65.5%, while the number of those reporting the use of ABHR remained high (81.2%).

### 3.3 Key indicators of the intervention

The intervention had many levels of participation across different professional categories. Non-medical staff had the highest participation rate in the training sessions (25.7%), followed by manual workers (15.1%) and medical staff (14.7%). Some departments, such as the intensive care unit (50% participation among physicians) and the dental department (50% among support staff), had better engagement. In addition, 500 liters of hand sanitizer and 36 awareness posters were distributed throughout the various departments, with the quantities allocated based on individual needs. The detailed information concerning the intervention is shown in Table 1.

**Table 1:** Indicators of the intervention.

Departments	Services	Training session attendance rate			Number of posters distributed	Quantity of PHA distributed per month
		Medical staff	Paramedical staff	Workers		
Medical unit	Pulmonology	3/10 (30%)	7/21 (33,3%)	0/2 (0%)	4	11 L
	Infectious diseases	0/3 (0%)	4/9 (44,4%)	0/2 (0%)	2	31 L
	Dentistry	0/6 (0%)	2/4 (50%)	1/2 (50%)	1	7 L
	Internal medicine	2/12 (16.6%)	6/19 (31.5%)	1/3 (33.3%)	3	24 L
	Hemodialysis	0/4 (0%)	3/11 (27.2%)	1/3 (33.3%)	0	26 L
	Pediatrics	3/20 (0.15%)	25/65 (38.4%)	1/4 (25%)	6	60 L
	Cardiology	2/10 (20%)	10/25 (40%)	1/4 (25%)	2	43 L
	Gastroenterology	2/7 (28.5%)	4/15 (26.6%)	1/2 (50%)	1	26 L
	Psychiatry	3/15 (20%)	4/20 (20%)	0/7 (0%)	0	12 L
Gynecology	Gynecology	4/16 (25%)	20/85 (23.5%)	5/27 (18.5%)	5	68 L
Emergency	Medical emergencies	0/20 (0%)	4/25 (16%)	0/4 (0%)	1	31 L
	Surgical emergencies	4/22 (18.1%)	10/40 (25%)	0/4 (0%)	1	40 L
Intensive care unit	Intensive care unit (ICU)	6/12 (50%)	4/27 (14.8%)	0/2 (0%)	3	4 L
	Anesthesiology/Intensive care	1/10 (1%)	6/30 (20%)	0/7 (0%)	3	15 L
Surgical unit	Orthopedics	0/12 (0%)	6/53 (11.7%)	0/4 (0%)	1	21 L
	Gerenal surgery	1/16 (0.06%)	8/34 (23.5%)	2/6 (33.3%)	2	36 L
	E.N.T	0/7 (0%)	3/12 (25%)	0/2 (0%)	0	7 L
	Urology	0/8 (0%)	8/28 (28.5%)	0/1 (0%)	1	5 L
Total		31/210 (14.7%)	134/520 (25.7%)	13/86 (15.1%)	36	500 L

### 3.4 Assessment of HCW’S knowledge and compliance in pre- and post-intervention

#### 3.4.1 Assessment of knowledge of HH

The rates of correct answers did not change significantly between pre- and post-intervention regarding the main microbial source (34% vs. 40.3%; *p* = 0.30) and the recommended handwashing duration (41.8% vs. 49.3%; *p* = 0.29). There was a slight decline in the rate of correct answers regarding the mode of transmission (56% vs. 53.3%; *p* = 0.29), with 29.6% still believing that contaminated surfaces are the main source of contamination. However, misconceptions persist regarding the characteristics of alcohol-based hand rubs (ABHR), particularly regarding skin dryness (54% incorrect answers). The detailed results are presented in Table 2.

**Table 2:** Assessment of knowledge levels among healthcare workers before and after the intervention.

Period		Pre intervention			Post intervention			*P*
Professional category		MEDICAL	paramedical	Total	Medical	Paramedical	Total	
Prior training in hand hygiene within the last 3 years		51/120 (42.5%)	81/180 (45%)	132/300 (44%)	79/121 (65.3%)	147/224 (65.6%)	226/345 (65.5%)	**<10–3**
Common use of alcohol-based hand sanitizers for hand hygiene		90/120 (75%)	152/180 (84.4%)	242/300 (80.7%)	89/121 (73.6%)	191/224 (85.3%)	280/345 (81.1%)	0.19
Mode of cross-transmission of pathogens responsible for healthcare-associated infections (correct answer: healthcare workers’ hands)		75/120 62.50%	93/180 51.70%	168/300 56%	72/121 (59.50%)	112/224 (50.00%)	184/345 (53.30%)	0.29
The most common source of microorganisms responsible for HAIs (correct answer: microorganisms from the patient)		31/120 25.80%	71/180 39.40%	102/300 34.00%	51/121 42.10%	88/224 39.30%	139/345 40.30%	0.30
Characteristics of alcohol-based hand rub (ABHR) and handwashing	Faster	74/120 (61.70%)	114/178 (64.00%)	188/298 (63.40%)	88/120 (73.3%)	149/224 (66.5%)	237/344 (68.9%)	0.63
	No skin dryness	55/119 (46.20%)	77/173 (44.50%)	132/292 (45.20%)	61/119 (51.3%)	112/219 (51.1%)	173/338 (51.2%)	0.25
	More efficient	35/120 (29.20%)	60/180 (33.33%)	95/300 (31.70%)	38/121 (31.4%)	79/224 (35.3%)	117/345 (33.9%)	0.50
	No recommendation to use both techniques	46/119 (38.7%)	74/178 (41.6%)	120/297 (40.40%)	51/121 42.10%	90/222 (40.50%)	141/343 (41.1%)	0.71
Duration of hand sanitizer application (40 s)		55/120 (45.80%)	70/179 (39.1%)	125/299 (41.8%)	63/121 (52.1%)	103/223 (46.2%)	166/344 (49.3%)	0.29
Situations to avoid as they could promote microbial colonization of the hands	Jewelry wear	119/120 (99.2%)	173/180 96.1%	292/300 (97.3%)	121/121 (100%)	219/224 (97.8%)	340/345 (98.6%)	0.18
	Damaged skin	91/115 (79.1%)	121/174 (69.5%)	212/289 (73.4%)	98/119 (82.4%)	165/222 (74.3%)	263/341 (77.1%)	0.21
	Artificial nails	112/119 (94.1%)	159/178 (88.8%)	271/298 (90.9%)	115/119 (96.6%)	198/222 (89.2%)	313/341 (91.8%)	0.25
	Protective lotion cream	98/115 (85.2%)	133/174 (76.4%)	231/289 (79.9%)	101/118 (85.6%)	179/217 (82.5%)	280/335 (83.6%)	0.14

#### 3.4.2 Assessment of compliance

##### 3.4.2.1 Overall compliance

Overall compliance increased from 19.9% (296/1484 opportunities) to 23.6% (348/1475 opportunities) after the intervention (*p* < 10^−3^) (Table 3).

**Table 3:** Assessment of hand hygiene compliance among healthcare workers before and after the intervention.

	Pre intervention			Post intervention			
	HH* opportunities n	Compliance *n* (%)	CI**95%	HH* opportunities n	Compliance *n* (%)	CI 95%	*p*
Total	1,484	296 (19.9)	18.9–20.9	1,475	348 (23.6)	21.4–25.7	**0.01**
Professional category							
Medical staff	419	94 (22.4)	18.4–26.4	444	121 (28.5)	24.3–32.7	0.06
Paramedical staff	853	179 (20.9)	18.1–23.6	838	196 (23.4)	20.5–26.2	0.38
Support staff	212	23 (10.8)	6.6–14.9	212	31 (14.6)	9.8–19.3	0.27
Department							
Medical unit	674	182 (27)	23.6–30.3	664	203 (30.6)	27.0–34.1	0.28
Intensive care unit	144	40 (27.8)	20.4–35.1	145	45 (31)	23.4–38.5	0.58
Gynecology	216	17 (7.9)	4.3–11.5	216	26 (12)	7.6–16.3	0.16
Surgical unit	234	42 (18)	13.0–22.9	270	51 (19.9)	15.1–24.6	0.35
Emergency room	216	15 (7)	3.5–10.4	180	23 (12.8)	7.9–17.6	0.19

Quality of hand hygiene practices							
	HH* opportunities n	Rate of high-quality actions *n* (%)	CI 95%	HH* opportunities n	Rate of high-quality actions *n* (%)	CI 95%	*p*
Total	296	64 (21.6)	16.9–26.2	348	93 (26.8)	22.1–31.4	0.15
Professional category							
Medical staff	94	21 (22.3)	13.8–30.7	121	29 (24)	16.3–31.6	0.90
Paramedical staff	179	38 (21.2)	15.2–27.1	196	57 (29.2)	22.8–35.5	0.10
Support staff	23	5 (21.7)	4.8–38.5	31	7 (22.6)	7.8–37.3	0.79
Department							
Medical unit	182	28 (15.4)	10.1–20.6	203	28 (13.9)	9.1–18.6	0.78
Intensive care unit	40	11 (27.5)	13.6–41.3	45	18 (40)	25.6–54.3	0.32
Gynecology	17	7 (41.2)	17.8–64.6	26	12 (46.2)	27.0–65.3	0.99
Surgical unit	42	9 (21.4)	8.9–33.8	51	24 (47.1)	33.4–60.8	**0.01**
Emergency room	15	9 (60)	35.2–84.7	23	11 (47.8)	27.3–68.2	0.59

Respecting the prerequisites							
	HH* opportunities n	Optimal opportunity rate *n* (%)	IC95%	HH* opportunities n	Optimal opportunity rate *n* (%)	IC95%	*p*
Total	1,484	552 (37.2)	34.8–39.6	1,475	524 (35.5)	31.1–37.9	**0.01**
Professional category							
Medical staff	419	177 (42.2)	37.5–46.9	444	167 (39.4)	34.9–43.9	0.06
Paramedical staff	853	280 (32.8)	29.7–35.9	838	289 (34.4)	31.2–37.6	0.38
Support staff	212	95 (44.8)	38.2–51.4	212	68 (32.1)	25.9–38.3	0.27
Department							
Medical unit	674	191 (28.3)	24.9–31.7	664	151 (22.7)	19.6–25.8	0.28
Intensive care unit	144	89 (61.8)	53.9–69.7	145	96 (66.2)	58.6–73.8	0.58
Gynecology	216	112 (51.9)	45.3–58.5	216	119 (66.1)	59.9–72.3	0.16
Surgical unit	234	61 (26.1)	20.5–31.7	270	56 (25.9)	20.8–31.1	0.35
Emergency room	216	99 (45.8)	39.2–52.4	180	102 (37.8)	30.8–44.8	0.19

*HH: hand hygiene. **CI: confidence interval.

Bold font indicate significant of *p* values.

##### 3.4.2.2 Compliance by professional category

The rate of correct answers increased in all three staff categories, remaining highest among physicians (from 22.4 to 28.5%, *p* = 0.06), followed by paramedical staff (20.9 to 23.4%, *p* = 0.38) and then manual workers (10.8 to 14.6%, *p* = 0.27).

##### 3.4.2.3 Compliance by department

Analysis by department shows an improvement in hand hygiene compliance without reaching statistical significance. The highest rates were observed in the intensive care unit (27.8 to 31.0%) and the medical ward (27.0 to 30.6%). The surgical ward showed moderate compliance (18.0 to 19.9%). However, rates remained low in the emergency department (7.0 to 12.8%) and in gynecology (7.9 to 12.0%), despite a slight increase.

##### 3.4.2.4 Compliance by indication

The analysis by indication shows that hand hygiene compliance is higher after contact with the patient (indication 4: 30.8 to 34.3%) and after a risk of exposure to bodily fluids (indication 3: 22.3 to 26.0%). Intermediate rates were observed before an aseptic procedure (indication 2: 16.1 to 21.2%) and before touching the patient (indication 1: 13.4 to 16.6%). Compliance remained lowest after contact with the patient’s environment (indication 5: 11.1 to 15.6%).

##### 3.4.2.5 Techniques used

Regarding the techniques used, handwashing was slightly more frequent before contact with the patient (53.0%) than after it (50.8%). Conversely, alcohol-based hand rub was used proportionally more often after (49.2%) than before (47.0%) contact with the patient. Overall actions of alcohol-based hand rub increased slightly, from 47 to 49.2% of actions.

##### 3.4.2.6 Quality of action

The rate of optimal quality actions increased from 21.6 to 26.8%, but 66.9% of the actions did not meet the recommended duration or steps.

##### 3.4.2.7 Compliance with prerequisites

Compliance with prerequisites remained stable: 37.2% of opportunities with optimal compliance before the intervention vs. 35.5% after the intervention. Compliance with the prerequisites regarding fingernails was the highest (92.7%), followed by jewelry (80.1%) and clothing (47.5%).

## 4 Discussion

### 4.1 Assessment of knowledge

Most articles discussed the overall knowledge score before and after the intervention, which increased in many studies (12) but some elements required more attention, such as the mode of transmission. In fact, the correct response rate to this question in our study was 56% pre-intervention and 53% post-intervention. A study conducted between January 2019 and December 2023 at Hedi Chaker and Habib Bourguiba hospitals in the Sfax governorate, Tunisia, reported an increase in the rate of correct responses from 67 to 84% (13). This rate was also around 69% in the study conducted at Sahloul Hospital (8) and 78.6% in the study conducted in Bamako (14). This slight decrease may be due to confusion between contact transmission and the fact that 29.6% of respondents stated that contaminated surfaces are the main mode of transmission, meaning they believe germs are acquired by touching contaminated surfaces.

Similarly, perceptions of the effectiveness and adverse effects of alcohol-based hand rub (ABHR) remain influenced by preconceived ideas: nearly half of healthcare workers still believe that ABHR dries the skin more than simple handwashing, and only a third recognize its superiority in terms of effectiveness. These misconceptions explain the minimal improvement in ABHR use: 49.2% after vs. 47.0% before. This was similar to the study conducted in Ethiopia (15). However, this rate remains lower than the 65.4% rate of participants in the study conducted at Sahloul Hospital (8) and the 74.8% rate in the Bamako study (14).

### 4.2 Compliance

Overall adherence to hand hygiene increased from 19.9% to 23.6%, a modest but significant improvement. These results remain within the range reported in developing countries (20%) (12, 16) and more specifically in Tunisian studies such as those conducted in Sousse and Sfax (8, 13). However, this rate was significantly lower than data from high-income countries (>40%) (17–20).

Compared to experiences where the multimodal model has led to significant progress, the impact in our study remains limited, suggesting persistent structural and organizational obstacles. Indeed, there was no visible improvement in terms of resources. In fact, the availability of essential resources such as alcohol-based hand rubs, soap, and functioning sinks remained unchanged, and in some cases, stock-outs persisted. The absence of wide-scale feedback of the findings, along with limited motivation among healthcare professionals, also hindered the overall effectiveness of the intervention.

The analysis by professional category shows a more marked improvement among doctors (22.4 to 28.5%) and paramedics (20.9 to 23.4%), while manual workers remain less involved (10.8 to 14.6%). These disparities are consistent with the heterogeneous results observed in the literature, where some studies reported better compliance among nurses (16, 19) and others among doctors (18).

Regarding the indications for hand hygiene, the highest compliance rates are observed after exposure to biological fluids (22.3 to 26%) and after contact with the patient (30.8 to 34.3%) (18).

On the other hand, “before” indications (before contact or before performing an aseptic procedure) remain largely neglected. This trend, widely documented in international studies, reflects a focus on protecting the healthcare worker rather than the patient, and underscores the need for a paradigm shift.

### 4.3 Technique and quality

The use of alcohol-based hand rub accounted for nearly half of all hand hygiene actions, with a slight increase (47 to 49.2%). This proportion is encouraging compared to some African studies [13% in Kisangani (21)], but lower than others [72% in Bamako (14)]. Therefore, the use of alcohol-based hand rub needs to be further promoted, particularly through improved access to products and by addressing misconceptions.

Compliance with basic hygiene practices (regarding nails, jewelry, and attire) is generally satisfactory, but the rate of optimal adherence remains low (37.2 to 35.5%). The quality of hand hygiene practices, assessed according to the WHO-recommended steps and duration, remains insufficient: only one quarter of the procedures were performed correctly, echoing the findings of several Maghreb and international studies.

## 5 Strength and limitation of the study

### 5.1 Advantages

This study provides valuable local evidence on the feasibility and impact of implementing the WHO multimodal strategy, showing a modest but significant improvement in hand hygiene compliance, especially among physicians and in intensive care which was one of the rare studies that showed this disparity.

### 5.2 Limits

The impact remained limited due to persistent structural barriers, insufficient motivation, and lack of large-scale feedback, with no significant improvement in resource availability.

## 6 Conclusion

The implementation of the WHO multimodal strategy at Ibn El Jazzar Hospital led to a modest but significant improvement in hand hygiene compliance, particularly among physicians and in intensive care. However, persistent gaps in knowledge, practices, and resource availability highlight the need for sustained efforts, continuous training, and stronger organizational support to achieve meaningful progress in infection prevention and control.

## Data Availability

The raw data supporting the conclusions of this article will be made available by the authors, without undue reservation.

## References

[1] World Health Organization. Global report on infection prevention and control 2024. Available online at: https://www.who.int/publications/i/item/9789240103986 (Accessed November 29, 2024).

[2] Lotfinejad N Peters A Tartari E Fankhauser-Rodriguez C Pires D Pittet D. Hand hygiene in health care: 20 years of ongoing advances and perspectives. Lancet Infect Dis. (2021) 21:e209–21. doi: 10.1016/S1473-3099(21)00383-2 34331890

[3] A guide to the implementation of the WHO multimodal hand hygiene improvement strategy. Available online at: https://www.who.int/publications-detail-redirect/a-guide-to-the-implementation-of-the-who-multimodal-hand-hygiene-improvement-strategy (Accessed February 9, 2009).

[4] Enqeute Nationale de Prevalence NOSOTUN 2012 Enquete Nationale. Disponible sur le site officiel du ministère de santé. (2012). Available online at: https://santetunisie.rns.tn/fr/

[5] Baklouti M Ben Hmida M Ben Ayed H Ben Jmeaa M Trigui M Trabelsi B . Health-care associated infections in the two university hospitals of southern Tunisia: a point prevalence survey. J Prev Med Hyg. (2025) 66:E94–E101. doi: 10.15167/2421-4248/jpmh2025.66.1.3397, 40756200 PMC12312722

[6] Ben Salem K El Mhamdi S Letaief M Bchir M Soltani MS. Epidemiological profile of health-care-associated infections in the central-east area of Tunisia. East Mediterr Health J 17 485–489 2011. Available online at: https://iris.who.int/handle/10665/118646 21796965

[7] Bouafia N Bougmiza I Bahri F Letaief M Astagneau P Njah M. Ampleur et impact des évènements indésirables graves liés aux soins: étude d’incidence dans un hôpital du Centre-Est tunisien. Pan Afr Med J 2013; 16. Available online at: http://www.panafrican-med-journal.com/content/article/16/68/full/10.11604/pamj.2013.16.68.1161 PMC3976656 24711868

[8] Thabouti M. L’hygiène des mains au CHU Sahloul: Evaluation des pratiques, des ressources et des connaissances des professionnels de la santé selon les recommandations de l’OMS. Sousse, Tunisie: Faculté de Médecine Ibn El Jazzar 2016

[9] Belhadj N. Implementation of the multimodal strategy of the who for hand hygiene improvement in sahloul university hospital: sustainability and challenges. Sousse Tunisie: Faculté de médecine ibn el jazzar (2023).

[10] Hygiène des mains: Quand et comment – Dépliant World Health Organization WHO (2019). Available online at: https://medbox.org/document/hygiene-des-mains-quand-et-comment-depliant

[11] The WHO multimodal hand hygiene improvement strategy—WHO guidelines on hand hygiene in health care—NCBI bookshelf. (2009). Available online at: https://www.ncbi.nlm.nih.gov/books/NBK144032/

[12] Yousef RHA Salem MR Mahmoud AT. Impact of implementation of a modified World Health Organization multimodal hand hygiene strategy in a university teaching hospital. Am J Infect Control. (2020) 48:249–54. doi: 10.1016/j.ajic.2019.07.019 31601445

[13] Ben Jmaa M Ben Hmida M Ben Ayed H Maamri H Trigui M Ortuño-Gutiérrez N . From knowledge to practice: The effect of multimodal strategies on hand hygiene improvement in Tunisia. Trop Med Infect Dis 11 juin. (2025) 10:162. doi: 10.3390/tropicalmed10060162 40559729 PMC12197701

[14] Camara N. Mise en place de la stratégie multimodale de l’OMS pour la promotion de l’hygiène des mains: Etat des lieux du Département de Gynécologie-Obstétrique du CHU Gabriel TOURE. 2012; Available online at: https://library.adhl.africa/handle/123456789/9182

[15] Pfäfflin F Tufa TB Getachew M Nigussie T Schönfeld A Häussinger D . Implementation of the WHO multimodal hand hygiene improvement strategy in a university hospital in Central Ethiopia. Antimicrob Resist Infect Control. (2017) 6:3. doi: 10.1186/s13756-016-0165-9 28070310 PMC5217264

[16] Arntz PRH Hopman J Nillesen M Yalcin E Bleeker-Rovers CP Voss A . Effectiveness of a multimodal hand hygiene improvement strategy in the emergency department. Am J Infect Control. (2016) 44:1203–7. doi: 10.1016/j.ajic.2016.03.017 27160981

[17] Casas I Castellà L Gimenez M Pulido A Sopena N Ciércoles A . Impact of a multimodal intervention on compliance with hand hygiene among health care workers of a tertiary hospital. Medicina Clínica (English Edition). (2022) 159:426–31. doi: 10.1016/j.medcli.2021.12.018 35210097

[18] Farhoudi F Sanaei Dashti A Hoshangi Davani M Ghalebi N Sajadi G Taghizadeh R. Impact of WHO hand hygiene improvement program implementation: a quasi-experimental trial. Biomed Res Int. (2016) 2016:1–7. doi: 10.1155/2016/7026169, 27999811 PMC5141532

[19] Allegranzi B Gayet-Ageron A Damani N Bengaly L McLaws ML Moro ML . Global implementation of WHO’S multimodal strategy for improvement of hand hygiene: a quasi-experimental study. Lancet Infect Dis. (2013) 13:843–51. doi: 10.1016/S1473-3099(13)70163-4 23972825

[20] Mu X Xu Y Yang T Zhang J Wang C Liu W . Improving hand hygiene compliance among healthcare workers: an intervention study in a Hospital in Guizhou Province, China. Braz J Infect Dis. (2016) 20:413–8. doi: 10.1016/j.bjid.2016.04.009, 27351752 PMC9425469

[21] Longembe EB Kitronza PL. Compliance with hand-hygiene practice in the general reference hospitals of the city of Kisangani, Democratic Republic of the Congo. Pan Afr Med J. (2020) 35:57. doi: 10.11604/pamj.2020.35.57.18500, 32537061 PMC7266366

